# Gonadal transcriptomes associated with sex phenotypes provide potential male and female candidate genes of sex determination or early differentiation in *Crassostrea gigas*, a sequential hermaphrodite mollusc

**DOI:** 10.1186/s12864-021-07838-1

**Published:** 2021-08-09

**Authors:** Coralie Broquard, Suwansa-ard Saowaros, Mélanie Lepoittevin, Lionel Degremont, Jean-Baptiste Lamy, Benjamin Morga, Abigail Elizur, Anne-Sophie Martinez

**Affiliations:** 1 Normandie University, UNICAEN, CNRS, BOREA, 14000 Caen, France; 2 Laboratoire de Biologie des Organismes et Ecosystèmes Aquatiques (BOREA), Université de Caen Normandie, MNHN, SU, UA, CNRS, IRD, Esplanade de la Paix, CS 14032, 14032, Cedex 05, Caen, France; 3 Ifremer, RBE-SG2M-LGPMM, La Tremblade, France; 4 Department of Anatomy, Faculty of Science, Mahidol University, Bangkok, Thailand; 5 Genecology Research Centre, University of the Sunshine Coast, Sippy Downs, Queensland Australia

**Keywords:** RNA-Seq, Gonad transcriptome, Early sex differentiation, Sex determination, Irregular hermaphrodite, Oyster

## Abstract

**Background:**

In the animal kingdom, mollusca is an important phylum of the Lophotrochozoa. However, few studies have investigated the molecular cascade of sex determination/early gonadal differentiation within this phylum. The oyster *Crassostrea gigas* is a sequential irregular hermaphrodite mollusc of economic, physiological and phylogenetic importance. Although some studies identified genes of its sex-determining/−differentiating pathway, this particular topic remains to be further deepened, in particular with regard to the expression patterns. Indeed, these patterns need to cover the entire period of sex lability and have to be associated to future sex phenotypes, usually impossible to establish in this sequential hermaphrodite. This is why we performed a gonadal RNA-Seq analysis of diploid male and female oysters that have not changed sex for 4 years, sampled during the entire time-window of sex determination/early sex differentiation (stages 0 and 3 of the gametogenetic cycle). This individual long-term monitoring gave us the opportunity to explain the molecular expression patterns in the light of the most statistically likely future sex of each oyster.

**Results:**

The differential gene expression analysis of gonadal transcriptomes revealed that 9723 genes were differentially expressed between gametogenetic stages, and 141 between sexes (98 and 43 genes highly expressed in females and males, respectively). Eighty-four genes were both stage- and sex-specific, 57 of them being highly expressed at the time of sex determination/early sex differentiation. These 4 novel genes including Trophoblast glycoprotein-like, Protein PML-like, Protein singed-like and PREDICTED: paramyosin, while being supported by RT-qPCR, displayed sexually dimorphic gene expression patterns.

**Conclusions:**

This gonadal transcriptome analysis, the first one associated with sex phenotypes in *C. gigas*, revealed 57 genes highly expressed in stage 0 or 3 of gametogenesis and which could be linked to the future sex of the individuals. While further study will be needed to suggest a role for these factors, some could certainly be original potential actors involved in sex determination/early sex differentiation, like paramyosin and could be used to predict the future sex of oysters.

**Supplementary Information:**

The online version contains supplementary material available at 10.1186/s12864-021-07838-1.

## Background

Sex differentiation is a physiological process triggered by the activation of one/a few sex-determining gene(s). In mammals, sex is attributed to the presence of chromosomes and sex determination corresponds to the activation of a gene that is necessary and sufficient for testis or ovary determination (for review see [38]). This event sets in train a cascade of interactions of differentiatinggenes that, in the end, direct the differentiation into testis or ovary. In this paper sex determination and sex differentiation are considered together since we could not distinguish the sex-determining from the sex-differentiating genes.

In sequential hermaphrodites, sex determination occurs during the embryonic development, as in gonochoric species. Sequential hermaphrodites are also able to change sex during their adulthood years [4, 19, 49]. However, it is unclear if these sex changes are only sex reversals (differentiated cells able to switch developmental fate: hypothesis 1) or if they involve a new sex determination (undetermined germ cells recruited within the gonad to initiate a new course of differentiation: hypothesis 2) [19]. The second hypothesis draws on the results of studies carried out in fish (for review see [19]). It is also often implicitly considered in the few existing studies on sequential hermaphrodite molluscs by mentioning sex determination mechanisms in adults [13, 42, 56–58, 62, 64]. This paper will also be based on the second hypothesis.

Mollusca is a phylum of lophotrochozoa encompassing around 85,000 species [50]. Although the number of whole-genome sequencing studies on molluscan species, especially among bivalves and gastropods, have greatly increased over the last decade (for more information, see the Genome Online Database GOLD, https://gold.jgi.doe.gov), only a few of them work on the understanding of the molecular mechanisms of reproduction, especially of sex determination and/or differentiation [7, 8, 13, 18, 20, 22, 28–30, 42, 48, 54, 57, 58, 61, 62, 64]. Yet, information at the molecular level is necessary to understand the evolution of these physiological mechanisms amongst the animal kingdom, and also to control the sex of species of aquaculture interest.

Among molluscs, *Crassostrea gigas* is the main bivalve product, with 639,030 tons produced in 2017. This species is known as a sequential hermaphrodite [10, 15, 31] that displays both genetic and environmental sex determination [12, 24, 31, 33, 45, 52]. The time window of sex determination / early gonadal differentiation in adults *C. gigas* has been defined between the end of a cycle, when the animals are mature and after spawning, at the beginning of a new cycle (respectively stages 3 and 0 according to [6]), two stages presenting germinal stem/progenitor cells [14] which, under the impulse of sex-determining genes will initiate a new male or female gonadal differentiation [22, 45, 46, 51, 53]. However, at both stages, the future sex of the oysters is unknown, especially as the oyster is devoid of identifiable heteromorphic sex chromosomes. Few orthologs of classical genes involved in sex determination/differentiation have been identified by targeted or more recently by large-scale studies, such as Foxl2 (Forkhead Box L2), Fem (Feminizer), Wnt-4, Gata-4 (Globin Transcription Factor 4), Run, β-catenin, SoxH (sex-determining region Y box*-*containing H/30, Dsx/Dmrt1-like (Doublesex- and mab-3-related transcription factor 1) and SoxE (Sex-determining region Y box*-*containing E) ([20, 22, 45, 46, 51, 53, 63] and 2014 - *C. gigas* genome v9 GCA_000297895.1 [62];). Most of these genes can also be found in other bivalves, ecdysozoan or vertebrates, suggesting that sex-determining/−differentiating pathways share common genes among vertebrates and ‘invertebrates’ [42, 51, 52, 62, 64]. However, the role of most of those genes in molluscs still requires further investigations.

In *C. gigas*, while almost no functional approach is available, genome-wide molecular studies are very powerful tools to study sex determination/differentiation. They provide large-scale expression patterns, which can be associated to putative roles in these physiological processes. Thus, a microarray-based analysis [22] identified sex-related genes and highlighted the sexually dimorphic gene expression of Foxl2 at stage 3, as previously identified [45] as well as 511 genes expressed at stage 0. Later on, a genomic analysis on mature animals (stage 3) ([64]; *Crassostrea gigas* genome v9 GCA_000297895.1) allowed for the identification of two new homologs of sex-determining/−differentiating genes *(*Dsx/Dmrt1-like and SoxH*)* and Cg-FoxN5 (*Crassostrea gigas* Forkhead Box N5), all presenting a male-specific expression. More recently, a gonadal RNA-Seq analysis [62] highlighted some genes clustered in five expression profiles: (i)- highly expressed at stage 1 [such as Dsx/Dmrt1-like, rather highly expressed in males at stage 3 by Zhang et al. [64]]; (ii) - decreasing or increasing expression throughout the oogenesis and spermatogenesis; (iii) - specific expression in female gonads [such as FoxL2 and Malrd1 (MAM and LDL-receptor class A domain-containing protein 1-like)]; (iv) – higher expression in male gonads; and (v) increased expression throughout spermatogenesis [such as SoxH, Cg-Sh3kbp1 (*C. gigas* SH3 Domain Containing Kinase Binding Protein 1, lncRNA (LOC105321313; long non-coding RNA) and uncharacterized LOC105345697]. However, apart from a few rare disagreements on the expression patterns, most of these previous studies are limited as follows: (1) the scarcity of information on expression at stage 0, (2) the impossibility to associate expression patterns to the future sex as it is unknown in this sequential and irregular hermaphrodite species and (3) the lack of information about ploidy, while it is naturally variable and it is known to modify the oyster’s gametogenesis [20].

## Results

### Transcriptomic analysis

The gonadal transcriptome sequencing generated a total of 1,424,810,382 raw reads (https://www.ncbi.nlm.nih.gov/bioproject/PRJNA660750) (724,160,832 and 700,649,550 reads for “true females” and “true males”, respectively) (Table 1). The average Phred score Q30 varied from 96.20 to 97.98%. After trimming and quality filtering, 1,388,380,771 clean reads were successfully mapped to the *Crassostrea gigas* genome (genome v9; Assembly GCA_000297895.1 [63]; see section “Methods” for more details) with an average efficiency of 97.48% (ranging from 96.44 to 98.52%). Among the 26,101 genes from the reference genome, 10,061 were detected in the present gonad transcriptomes (from all samples sequenced).

**Table 1:** Summary statistics of *Crassostrea gigas* gonad transcriptome sequencing. Three biological replicates per stage (0, 1 and 3) and sex (F = female, M = male)

Stage	Sample identity	Mapped reads (count)	Mapped reads (%)	Average length of mapped reads (bp)	Average Phred score 20 (%)	Average Phred score 30 (%)
0	F1	103,017,875	96.91	140.15	99.4	96.7
	F2	92,804,237	96.44	140.75	99.45	96.94
	F3	105,541,018	96.82	141.33	99.66	97.08
	M1	84,283,633	96.44	140.50	99.4	96.88
	M2	68,444,100	97.07	140.10	99.63	97.25
	M3	76,120,564	97.51	139.14	99.49	97.33
1	F4	42,539,142	96.68	140.39	98.92	96.2
	F5	51,467,467	97.42	138.93	99.58	97.21
	F6	57,539,741	98.05	139.44	99.86	97.92
	M4	68,411,348	98.19	141.55	99.8	97.93
	M5	81,529,233	97.23	141.39	99.83	97.82
	M6	64,087,363	98.08	141.64	99.79	97.43
3	F7	75,387,566	98.31	141.85	99.81	97.78
	F8	104,268,494	97.61	142.49	99.89	97.98
	F8	72,514,064	98.52	142.85	99.77	97.62
	M7	86,192,288	97.85	142.01	99.87	97.87
	M8	68,983,407	97.89	142.32	99.73	97.5
	M9	85,249,231	97.71	142.14	99.78	97.73

**Fig. 1:**
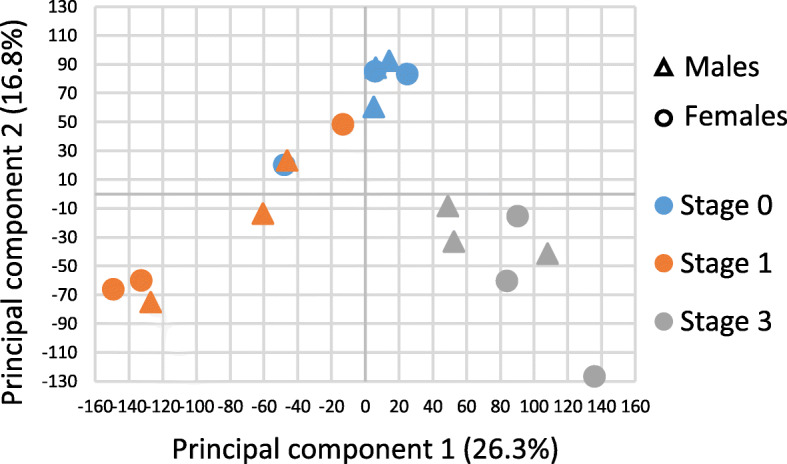
Scatter plot based on expression patterns of the 10,061 genes in the eighteen gonadal samples. Triangle symbols are for females and round symbols for males. Blue: stage 0; Orange: stage 1; Grey: stage 3

**Fig. 2:**
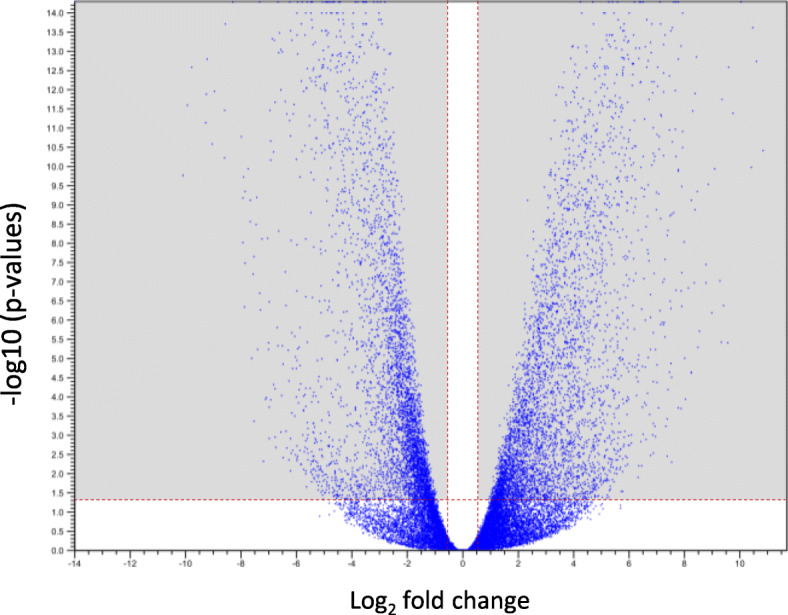
Volcano plot from the 10,061 genes expressed in the eighteen transcriptomes according to gametogenetic stages. Each point represents the average value of one gene in three replicate experiments. Horizontal red dotted line: -log_10_(0.05) = 1.30; vertical red dotted lines: log_2_ (1.5) = 0.58. The dots in the two shaded squares correspond to the significant values for which *p* < 0.05 and the fold change is higher than **|**1.5**|**

A principal component analysis (PCA) was performed on all 10,061 genes of the 18 gonads sampled to assess the homogeneity of the whole dataset and the degree of correlation of gene expression patterns at sex and gametogenic stages. The first two principal components explained 43.1% of the total variance. The scatter plot (Fig. 1) based on expression patterns of the expressed genes showed that transcriptional profiles are rather clustered by gametogenetic stage (determined by histology) than by sex (determined by long-term monitoring of sex phenotypes). A volcano plot used as a function of gametogenesis stages (Fig. 2) showed a good scattering of dots, thus demonstrating a significant differential gene expression according to gametogenetic stages.

### Differential gene expression analysis

**Fig. 3:**
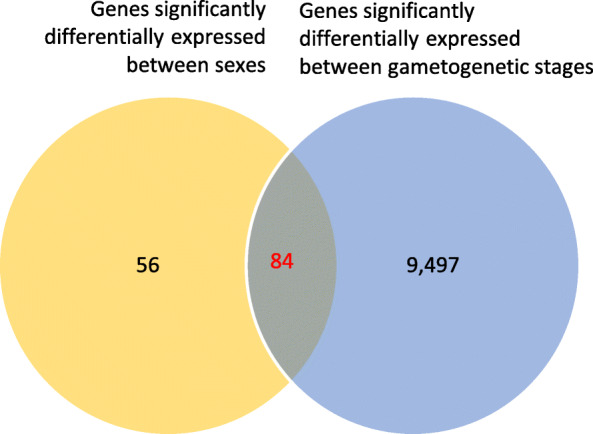
Venn diagram within gonad transcriptome for genes significantly differentially expressed between gametogenetic stages and sexes. 9582 genes significantly differentially expressed between gametogenetic stages are indicated in blue. 141 genes significantly differentially expressed between sexes are indicated in orange

A Differential Expression Analysis (DEA) was conducted in order to identify sex- and stage-specific gene expression patterns. It revealed a total of 9723 genes (96.64% of the 10,061 gonadic genes) significantly differentially expressed either between gametogenetic stages (9582 genes; 95.24%) or males and females (141 genes; 1.40%) with a FDR < 0.05 (Fig. 3). Among genes with a sex-specific expression, 98 (69.50%) showed a significantly higher expression in females and 43 (30.50%) in males. When data from both DEA were coupled, 84 genes (0.86%) were significantly differentially expressed between gametogenetic stages and sexes (Additional file 1).

These genes were classified by stage with 13, 27 and 44 genes showing a higher differential expression at stage 0, 1 and 3, respectively, and by sex with 46 and 38 genes showing a higher differential expression in females and males respectively (Table 2). Fifty-seven of them (67.9%) were significantly highly expressed in one sex during the time-window of sex determination/early gonadal differentiation which extends from stage 3 of 1 cycle to stage 0 of the next cycle.

**Table 2:** Distribution of the 84 genes significantly differentially expressed between males and females and gametogenetic stages. Percentages (%) of genes are in brackets

Sex	Stage 0	Stage 1	Stage 3	Total
**Female**	12	16	18	46 (54.8%)
**Male**	1	11	26	38 (45.2%)
**Total**	13 (15.5%)	27 (32.1%)	44 (52.4%)	84

**Fig. 4:**
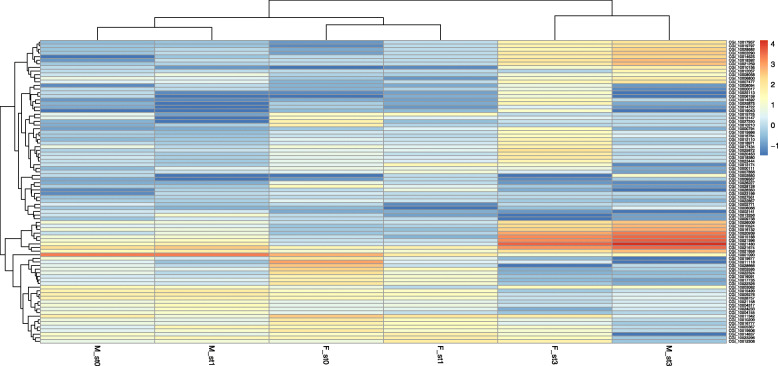
Heat map of 84 genes resulting from the DEA. Hierarchical clustering obtained using Pearson’s correlation on the 84 genes differentially expressed between males and females and between gametogenetic stages. Each column represents a stage associated to a sex, each row represents a gene. The clustering branch indicates similarity between genes or samples. The color scale represents the normalized log values (log_10_ FPKM) and indicates the different expression levels of transcripts, with shades of orange for higher gene expressions and shades of blue for lower gene expressions. st0: stage 0; st1: stage 1; st3: stage 3; M: male; F: female

The heat map based on log_10_ (FPKM; Fragments Per Kilobase of transcript per Million mapped reads) of DEA (Fig. 4) revealed that hierarchical clustering grouped the genes into five clusters and samples into three clusters: gonads at early female gametogenetic stage (F_st0 and F_st1), at early male gametogenetic stage (M_st0 and M_st1) and gonad at late gametogenetic stage regardless of gender (F_st3 and M_st3). The expression profiles appeared more homogeneous between the first two stages of a sex than between the two sexes of the same stage.

### Gene ontology analysis

**Fig. 5:**
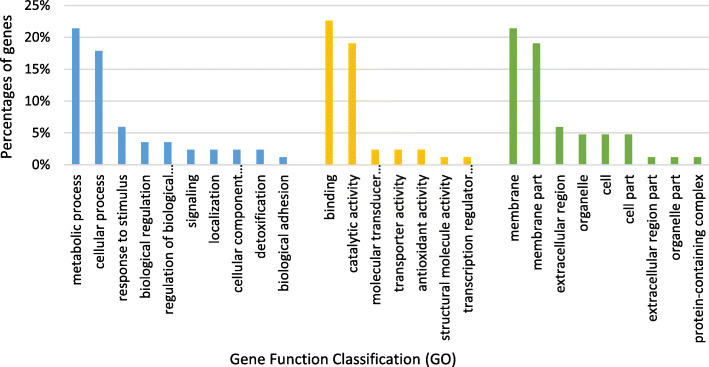
Gene ontology (GO) annotation (at level 2) of sex- and stage-biased genes of *C. gigas*. Distribution (%) of GO terms among the sex- and stage-biased genes. GO ontologies are represented as general function categories. Blue: biological process; yellow: molecular functions; green: cellular components

A gene ontology (G0) annotation was performed at level 2 among the 84 genes resulting from the DEA (Fig. 5). A total of 47 genes (56%) had eggNOG (evolutionary genealogy of genes: Non-supervised Orthologous Groups) assignments and 49 genes (58.3%) were assigned with at least one GO term. Within the “biological process” category, the main GO terms were grouped in metabolic (21%) and cellular processes (18%). Binding (23%) and catalytic activity (19%) were predominantly assigned to “molecular functions”. In the category “cellular components”, the largest proportion of GO terms referred to the membrane (40%). A GO enrichment analysis was conducted to identify the over-representation of GO terms based on sex- and stage-biased differentially expressed genes. However, no enrichment of GO terms was found.

### Expression patterns of candidate genes identified from the DEA, measured by real-time qPCR during the entire gametogenetic cycle

RT-qPCR were performed on seven genes (Table 3) selected from the 84 relevant ones (in addition to *FoxL2*; GenBank Accession Number: FJ768956.1) in order to support the RNA-Seq analysis and to explore their temporal expression during an entire gametogenetic cycle (stages 0, 1, 2 and 3 for males and females). They were chosen because, by RNA-Seq, they exhibited a significantly higher expression during the sex-determining period (i.e. stages 0 or 3) which also revealed a dimorphism between sexes. In other words, these seven genes emerged from the DEA of the RNA-Seq as having a significantly different expression between gametogenetic stages (0 versus 1, 0 versus 3 or 3 versus 1 *ie* exhibiting a significant peak of expression in stage 0 or 3, the sex-determination period) and also between sexes (significantly higher in male or in female). According to these criteria and based on the literature, we selected (1) one gene significantly highly expressed in stage 0 female (Predicted: paramyosin), (2) three significantly highly expressed in stage 3 male (Trophoblast glycoprotein-like, Protein singed-like, Hypothetical protein) and (3) three significantly highly expressed in stage 3 female [protein PML-like (Protein ProMyelocytic Leukemia-like), MAM and LDL-Receptor protein 1-like, Gata protein 14-like]. FoxL2 was used as a gold standard. EF1α, with a STD of 0.72 and with a r of 0.861, was considered as stable and was therefore chosen as housekeeping gene. As expected, for each primer pair, no DNA was amplified in the negative controls (sterile water and non-RT DNA) (Results not illustrated).

**Table 3:** Seven genes identified in the *C. gigas* transcriptome and selected for RT-qPCR

Gene name	Due to stage FDR ***p***-value	Due to sex FDR ***p***-value	Blasted in the oyster’s genome to	Blast e-value
**CGI_10,006,800**	4,44E-11	2,85E-04	Trophoblast glycoprotein-like	0.0
**CGI_10,028,666**	2,47E-14	1,51E-04	PREDICTED: paramyosin	0.0
**CGI_10,016,132**	0,00E+ 00	1,51E-04	Protein singed-like	0.0
**CGI_10,026,009**	0,00E+ 00	3,66E-02	Hypothetical protein	0.0
**CGI_10,018,971**	1,05E-02	8,93E-04	Protein PML-like	0.0
**CGI_10,008,094**	5,88E-05	8,79E-05	GATA zinc finger domain-containing protein 14-like	0.0
**CGI_10,025,872**	3,34E-03	1,62E-03	MAM and LDL-receptor class A domain-containing protein 1-like	0.0

**Fig. 6:**
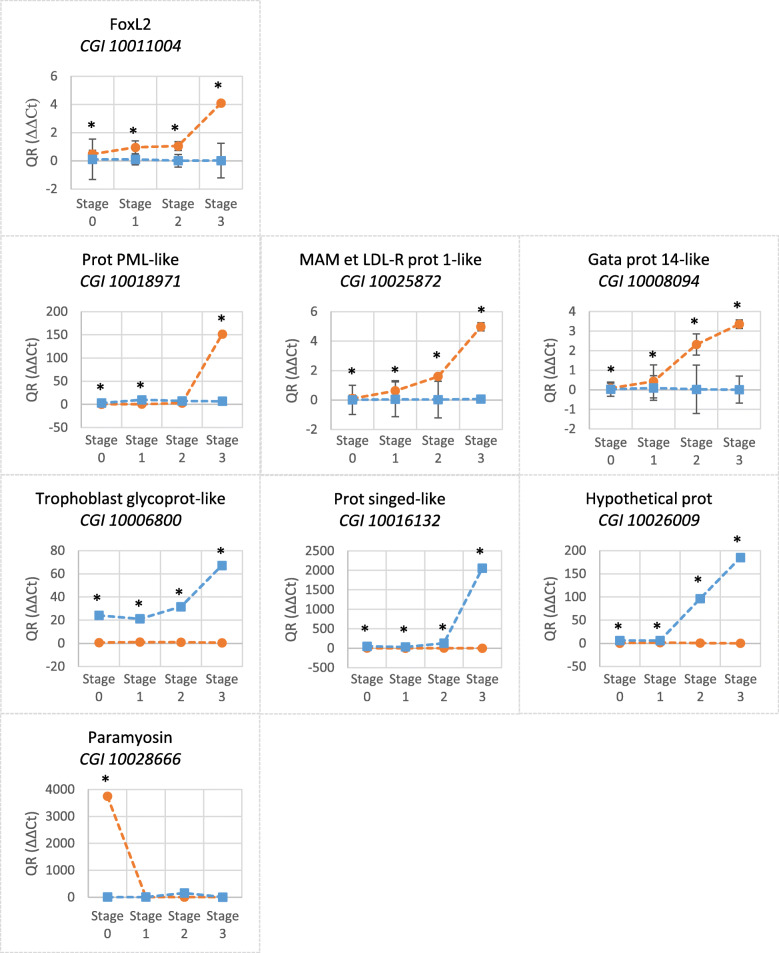
Expressions of eight relevant genes in RT-qPCR in gonads along the gametogenetic cycle. FoxL2 was taken as a gold standard. Females: orange lines, males: blue lines. *N* = 3 for each stage (stages 0, 1, 2 and 3) and sex. QR: Relative Quantity. Values are means + SEM of triplicates. Stars indicate significant (*p* < 0.05) differences in gene expression at one gametogenetic stage, between males and females

These seven genes exhibited a dimorphic expression by RT-qPCR, especially more significantly at stage 3 (Fig. 6 and Additional file 2). Four of them increased their expression over the course of gametogenesis in females (by a factor of 3 to 150; CGI 10011004-FoxL2, CGI 10018971-Protein PML-like, CGI 10025872-MAM and LDL-receptor class A domain-containing protein 1-like and CGI 10008094-GATA zinc finger domain-containing protein 14-like) and four in males (by a factor of 40 to 2000; CGI 10006800-Trophoblast glycoprotein-like, CGI 10016132-Protein singed-like and CGI 10026009-Hypothetical protein). For the “female genes”, the expression of CGI 10011004 (FoxL2) and CGI 10018971 (Protein PML-like) sharply increased between stages 2 and 3, while the increase was more progressive for CGI 10025872 (MAM and LDL-receptor class A domain-containing protein 1-like) and CGI 10008094 (GATA zinc finger domain-containing protein 14-like). For the “male genes”, the expression sharply increased from stage 2 for CGI 10006800 (Trophoblast glycoprotein-like) and CGI 10016132 (Protein singed-like) and from stage 1 for CGI 10026009 (Hypothetical protein) onwards. One gene (CGI 10028666 - PREDICTED: paramyosin) was more highly expressed in females than in males at stage 0 (× 3900) and its expression collapsed from stage 1 onwards.

## Discussion

The aim of our study was to shed light on the sex-specific molecular events that take place during sex determination/early gonadal differentiation in oysters *C. gigas*. For this purpose, we identified, by RNA-Seq analysis, genes that were significantly differentially expressed between “true males” and “true females” at the time of these physiological processes. To be able to select, for the first time, these “true males” and “true females” in such a sequential irregular hermaphrodite, it was necessary to work on animals whose sex phenotypes have been known for several consecutive years and in particular oysters, which never changed sex all along the course of study. This allowed us to develop the experimental strategy discussed below.

### Experimental strategy: first association of molecular expression patterns over the entire time-window of sex determination/early gonadal differentiation to future sex phenotypes

The challenge was compounded: - (1) highlight relevant genes covering the entire sex-determining/early gonadal differentiating period, which extends from stage 3 of one gametogenetic cycle when animals are matures to stage 0, just after spawning when the animals start a new cycle [22, 45, 46, 51, 53]. - (2) link each molecular expression pattern to a sex phenotype, *i.e* to the most statistically likely future sex of the animal, while this future sex is usually impossible to determine because *C. gigas* is a sequential irregular hermaphrodite.

Previous studies carried out on this topic in *C. gigas* are scarce and rather focused on mature animals [22, 62, 64] than on oysters at stage 0 [22]. Moreover, the gene expression patterns were never associated with the future sex of the animals. The same limitation exists for the very few large-scale molecular studies on sequential hermaphrodite oysters *C. hongkongensis* [58] and *Pinctada margaritifera* [56, 57], conducted on animals whose future sex was unknown. These limits also exist for studies made in simultaneous hermaphrodites, such as *Argopecten purpuratus* [7]. In *Nodipecten subnosus*, Galindo-Torres et al. [28] worked on undifferentiated juvenile gonads without discriminating against future sexes. They therefore adopted a targeted approach, looking for known homologs or genes containing key words “sex determination/differentiation”, “development”, “germ line”, “spermatogenesis” or “oogenesis”.

In the present study on *C. gigas*, transcriptomes were made from gonads of individuals (i) at stages 0 and 3, the time-window of sex determination/early gonadal differentiation and at stage 1, right afterwards, for comparative purposes and (ii) identified as males and females that had not demonstrated a sex change during the first four years of their life [10]. The challenge was to assign the most likely future sex phenotype to each oyster in order to better explain the expression patterns, at stages 0 and 3, when the future sex cannot be determined. To this purpose, we worked on 4-year-old oysters for two main reasons: (i) the older the oysters get, the less they change sex and (ii) less than 3% of the 4-year-old oyster population changed sex [10]. To strengthen our results, we also made sure that oysters that went through the RNA-Seq pipeline were all diploids (results not shown), while it was never determined/mentioned in the papers cited above. Yet in *C. gigas,* ploidy can vary naturally [1] and it is also known to modify gametogenesis and transcriptomes [20].

### The safeguards of transcriptome quality

The clean reads obtained presented high values of Quality score Q20 (from 98.92 to 99.89%) and Q30 (from 96.20 to 97.98%). This quality of sequencing is similar, although superior, to that reported for other molluscan transcriptomes. Indeed, Yue et al. [62] obtained Q20 ranging from 94.74 to 98.14% for *C. gigas.* Chen et al. [13] reported Q30 of at least 89.46% for their eight transcriptomes of *Tegillarca granosa*. In mussels, the Q20 was equal to 98.31% for *Cristaria plicata* [48] and ranging from 97.36 to 97.67% for *Hyriopsis schlegelii* [54]. Finally, transcriptomes of the gastropod *Reishia clavigera* provided Q30 ranging from 88.02 to 90.90% [36]. The transcriptomes carried out in the present study had higher rates of alignment to *C. gigas* genome (from 96.44 to 98.52%) than the one found by Yue et al. [62] (from 57.07 to 68.37%). No information was mentioned by Zhang et al. [64] on this aspect. Finally, the DEA we performed showed 9723 differentially expressed genes (DEG) for the gonad transcriptomes, which is similar to the 9343 DEGs reported by Yue et al. [62] in the same species. A lower number (1937) of DEGs was found in the gonad transcriptomes of the oyster *Pinctada margaritifera* [56].

### Genes differentially expressed between sexes during the sex-determining/−differentiating time window in *C. gigas*

Our analysis provided a list of 57 genes significantly differentially expressed between gametogenetic stages and sexes, with a significantly higher expression during the sex-determination time-window (stages 0 and 3) (Table 2 and Additional File 1). Amongst the 13 genes highly expressed in stage 0, 12 could be associated with a future female sex and 1 with a male sex. In the same way, amongst the 44 genes highly expressed at stage 3, 18 could be associated with a future female sex and 26 with a male sex. To our knowledge, this is the first time that genes could be linked with the future sex in *C. gigas* or in a hermaphrodite mollusc, thanks to our experimental strategy (see 1st paragraph). Amongst all these genes, some could certainly be original potential actors involved in sex determination/early sex differentiation, but not all of them. Indeed, in stage 0, when the gonad restructures after spawning, among the physiological processes that take place, we can count for instance the regression of gonadal tubules and the reformation of the storage tissue associated to an increase of circulating haemocytes [6, 25]. The stage 3 is the last stage of gametogenesis before spawning. It corresponds to the mature reproductive stage where spermatids and spermatozoa in males or pre-vitellogenic and vitellogenic oocytes in females constitute the majority of the germ cells present in the tubules [6, 27]. However, at this stage, putative germinal stem cells are also present at the edge of the tubules [14] which, under the impulse of sex-determining genes will initiate a new male or female gonadal differentiation while animals are still mature and ready to spawn [45, 46, 51, 53]. Thus, further investigations will be necessary to precisely characterize the 57 factors we found and define their spatial and temporal expression in order to determine which ones are involved in the oyster’s sex determination or early differentiation.

The genes resulting from the DEA and studied by RT-qPCR were segregated on the basis of the gametogenetic stage presenting their highest expression. To our knowledge, most of them had not been previously identified in *C. gigas* or in other molluscs.

Among the statistically dimorphic genes, those expressed at mature stage (stage 3) highlighted two different patterns.

The first pattern showed a regular increase of expression following gametogenesis from stage 1 to stage 3, in males for CGI 10026009 (Hypothetical protein) and in females for CGI 10008094 (GATA zinc finger domain-containing protein 14-like) and CGI 10025872 (MAM and LDL-receptor class A domain-containing protein 1-like). The hypothetical protein (CGI 10026009) blasted with the mammalian protein TESK2 (Testis Associated Actin Remodelling Kinase 2), a testis-specific protein kinase 2 suggested to be involved in meiotic stages and/or early stages of spermiogenesis in rat [26]. Its expression in *C. gigas* is in line with such a role. The female-specific expression increasing over gametogenesis observed in *C. gigas* for CGI 10025872 (MALRD1, MAM and LDL-receptor class A domain-containing protein 1-like) was similar to the one obtained by RT-qPCR for the so called ovarian gene *Cg-MALRD1-like* by Yue et al. [62]. Although this factor is rather expressed in the digestive tract in mammals, Yue et al. [62], based on their results in *C. gigas* gonad, suggested that the Cg-Malrd1-like gene might mediate signal-cell interactions to activate the oogonia or oocyte DNA replication process. Concerning GATA zinc finger domain-containing protein 14-like (CGI 10008094), it blasted with the mammalian *Gata-4*. This latter protein is present in bipotential gonads of XX and XY mouse embryos and is markedly down-regulated shortly after the histological differentiation of the ovary, suggesting an involvement in early gonadal development and possibly sexual dimorphism [60]. Its role in *C. gigas* still needs to be elucidated. To conclude, a role of these genes in sex determination/early differentiation seems unlikely given their patterns of expression during gametogenesis, which do not show a significant peak of expression in stage 3 compared to earlier stages, neither in males nor in females.

The 2nd pattern observed in *C. gigas* in the present study showed a sudden peak of expression at stage 3, for males for CGI 10016132 (Protein singed-like) and to a lesser extent for CGI_10,006,800 (Trophoblast glycoprotein-like) and in females for CGI 10018971 (PML-like-protein) and CGI_10,011,004 (FoxL2), which is the gold standard which exhibits the same expression pattern as the one reported by Santerre et al. [53]. This female expression of gene encoding PML-like-protein is in line with previous studies. Indeed, in *C. elegans*, the PML protein (sub)-nuclear localization seems to be affected by SUMO (Small Ubiquitin-like Modifier), required for gonadal and uterine-vulval organogenesis [9]. In mice, the *PML* transcript isoform II was found in mature sperm and the isoforms I and II in oocytes, suggesting that mature gametes may carry the transcripts to the embryo [23]. In mice, once again, Hadjimichael et al. [32] showed that over-expression of the *PML* gene in embryonic stems cell lines delays cell differentiation, suggesting its essential role as regulator of stem cell pluripotency and somatic cell reprogramming. In *C. gigas*, in the current state of knowledge, it is impossible to discriminate, for the PML-like-protein, a role in late gametogenesis (spermiogenesis) or in very early events preparing the new cycle (sex determination/early sex differenciation), both occurring in mature gonads at stage 3. Now concerning the protein singed-like, whose gene is homologous to the mammalian *fascin*, it is required, in drosophila, for actin filament bundle formation in the cytoplasm of nurse cells during oogenesis [11]. In mice, Tubb et al. [59] reported a testis *fascin* (*FSCN3, Fascin Actin-Bundling 3*), specifically expressed in the elongated spermatids and remaining in mature spermatozoa. The expression found in the present work in *C. gigas* does not fit exactly with such localization as many elongated spermatids are already present at stage 2, but this gene expression only increased after this stage. The expression of CGI 10006800 (Trophoblast glycoprotein-like) although always dimorphic in favour of the males, also highlighted a peak of expression at stage 3. A homolog of the Trophoblast glycoprotein, called Waif1a (Wnt-​activated inhibitory factor 1a), was reported, in zebrafish and xenopus male embryos as well as in mammalian cells, to inhibit the canonical Wnt (Winnt)/β-catenin signalling involved in female sex-determination [37]. The clear male dimorphic expression of this gene in *C. gigas* is in line with its likely role in the inhibition of the Wnt/β-catenin signalling, notably at stage 3, during the sex determination/early differentiation time-window. To conclude, further investigations need to be carried out on these three genes to elucidate their role in *C. gigas* gonads. However, an involvement in sex determination/early differentiation cannot be ruled out considering their statistical dimorphic expression and their sudden and significant peak of expression in stage 3 compared to earlier stages. Foxl2, once again, appears as a very likely candidate for female sex determination in *C. gigas*.

Finally, one gene showed a peak of expression at stage 0 followed by a sharp decrease, CGI 10028666 (PREDICTED: paramyosin) in females only. In molluscs, paramyosin is a protein found in muscles [16]. In drosophila, it was suspected to up-regulate the expression of *Tra2* (*Transformer 2*) in males, outside the sex determination pathway, as an off-target effect [2]. In cephalobidae nematods and in *C. elegans*, paramyosin is a structural component of the female gonad which plays an essential role in ovulation [5, 47]. Both roles do not fit with the expression found in the present study, in females and at the very beginning of the gametogenesis. Interestingly, paramyosin of *Schistosoma mansoni* is homologous to *Taenia solium* antigen B, an immunogenic protein with anti-complement activity [40]. In vertebrates, a H-Y antigen is expressed in the heterogametic sex regardless of whether male or female. Its role, although still controversial, was notably suggested to be associated with a common denominator underlying the development of mammalian testes and avian ovaries which enhanced the growth rate of the dominant heterogametic gonad at a critical stage of development [44]. In mammals, such serologically detectable male antigens could also be associated with testis activity or spermatogenesis and may be antigenic when expressed in females [55]. In fish, some observations suggest that it may play a secondary role in primary sex determination but may well be very important for subsequent differentiation or re-differentiation [19]. Although it clearly appears that the role of paramyosin has to be elucidated in *C. gigas*, its female-specific expression at stage 0 during the oyster’s sex determination/early differentiation time-window points to a possible important role in this process.

## Conclusions

To conclude, 57 genes highly expressed in stage 0 or 3 of gametogenesis and which could be linked to the future sex of the individuals emerged from the present work. Several of them could certainly be original potential actors involved in sex determination/early sex differentiation, like PREDICTED: paramyosin (CGI 10028666), Trophoblast glycoprotein-like (CGI_10,006,800), protein Singed-like (CGI 10016132) and PML-like-protein (CGI 10018971), also studied here by RT-qPCR. Amongst them, some may have direct effects in the sex determination/early differentiation pathway and others could indirectly be associated with sex determination/ early differentiation time-window in *C. gigas*. However, because little information is available on most of these factors for *C. gigas* and other molluscs, further investigations will be essential to clarify their homologies, as well as their spatial and temporal expression and their roles in the oyster’s gonad.

## Methods

### Sampling and animal selection strategy

**Fig. 7:**
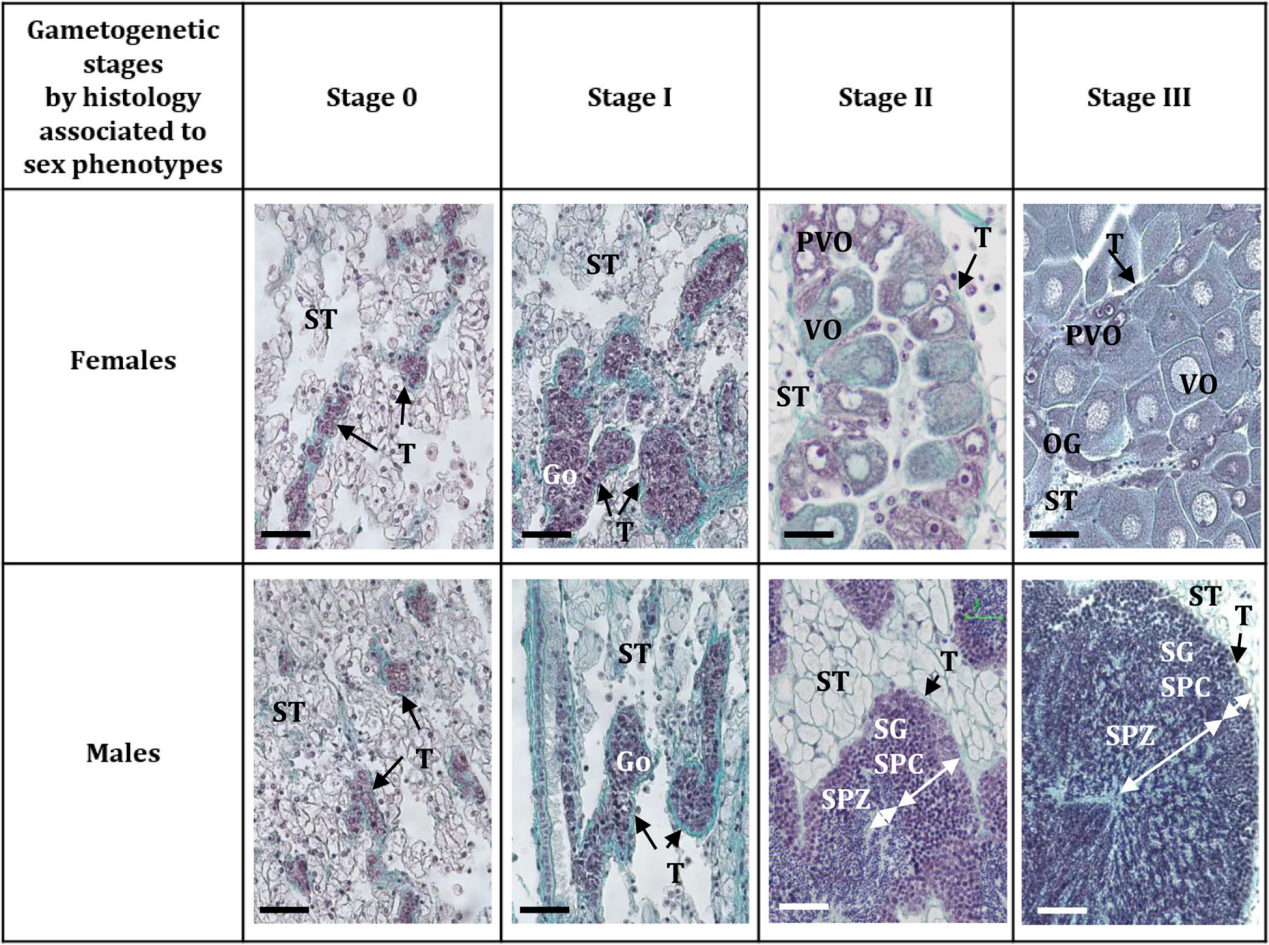
Description and histological illustrations of sex and gametogenetic stages used for RNA-sequencing and for RT-qPCR. Based on the criteria described by Heude-Berthelin et al. [34] and on the determination of sex phenotypes by Broquard et al. [10]. Stage 0, undifferentiated cells; stage 1, gonia proliferations; stage 2, gonia maturation; stage 3, ripe gonads with mainly spermatozoa and mature oocytes; some undifferentiated cells. Stages 0, 1 and 3 for RNA-sequencing; stages 0, 1, 2 and 3 for RT-qPCR. Go: gonia, OG: oogonia, PVO: pre-vitellogenic oocytes, SG: spermatogonia, SPC: spermatocytes, SPZ: spermatozoa, ST: storage tissue, T: gonadal tubule, VO: vitellogenic oocytes. Bars: 25 μm

The *C. gigas* oysters are from a same full-sib family produced at the Ifremer facilities from a wild broodstock in March 2013 (for more information see [3]). Seven thousand four hundred and eighty-eight oysters were monitored in this environment and sexed since then (for more information see [10]). Twenty-four of them were individually selected according to their sex phenotype based on the 4th previous years and on their gametogenetic stage. They were sampled in January, February and June 2018, and their gonads were collected for RNA extractions (snap frozen in liquid nitrogen) and histology (fixed in a Davidson’s fixative solution). Gills were also collected and conserved in 70% ethanol for flow cytometry analysis. This allowed us to verify that the chosen oysters were diploids, as ploidy can be very plastic in this species [41]. The gametogenetic stages were determined a posteriori based on the criteria described by Heude-Berthelin et al. [34]: Stage 0 (post-spawning undifferentiated cells), stage 1 (gonia proliferation), stage 2 (maturation; spermatogenesis or oogenesis) and stage 3 (ripe gonads before spawning; undifferentiated cells). Histologically, during one cycle, sex can traditionally only be determined from the end of stage 1 (therefore not in stage 0) and sex for the next cycle can never be predicted because *C. gigas* is a sequential irregular hermaphrodite. Thus, in order to be able to “discriminate between future males and future females”, an asset of our study was to work on oysters which had had no sex change over the 4 previous years, with the aim to assign a “very likely” future sex phenotype to these “true males” and “true females”. Indeed, two arguments support this choice: (i) the older the oysters get, the less they change sex; (ii) less than 3% of the 4-year-old oyster population changed sex ([10], see supplementary material). Hence, nine males and nine females were chosen at stages 0, 3 and 1 (3 individuals per stage) for RNA-Seq analysis. The first two stages correspond to the time-window of sex determination/early sex differentiation in this species [22, 45, 46, 51, 53] and the last one is for comparison (Fig. 7). Quantitative Real time PCR (RT-qPCR) were also performed on twenty-four animals (3 individuals per stage and per sex) covering the entire gametogenetic cycle, i.e. stages 0, 1, 2 and 3 (Fig. 7).

### RNA preparation, cDNA library construction and Illumina sequencing

The total RNA from 18 gonads (3 males and 3 females per stage; stages 0, 1 and 3) were individually extracted using TRI Reagent (Sigma®), treated with TURBO™ DNAse (Invitrogen) to remove genomic DNA and purified with Direct-zol™ RNA MiniPrep (Zymo Research) following the manufacturer’s protocol. RNA concentration, integrity and purity were assessed on agarose gels (1%) and using NanoDrop 2000 (Thermo Scientific), Qubit® 2.0 Fluorometer (Invitrogen) and Bioanalyzer 2100 (Agilent Technologies). Complementary DNA libraries were made from the total RNA of the 18 individual samples, nine per sex, conformed to the required purity criteria (A260/A230 and A260/A280 > 1.8) and quality levels (RNA Integrity Number, RIN > 8) for cDNA library preparation. Complementary DNA libraries were constructed by means of the LIGAN-PM platform (Lille, France) using TruSeq RNA Library Prep kit v2 (Illumina) in line with the manufacturer’s recommendations. Briefly, per library, approximately 1 μg of the total RNA sample was purified using oligo-dT beads, followed by a fragmentation with Elute Prime, Fragment Mix (Illumina). First-strand cDNA was generated by reverse transcription with First Strand Master Mix (25 °C for 10 min, 42 °C for 50 min and 70 °C for 15 min) and the synthesis of second-strand cDNA was performed in the presence of Second Strand Master Mix and dATP, dGTP, dCTP, dUTP mix (16 °C for 1 h). Purified fragmented cDNA was incubated in presence of End Repair Mix (30 °C for 30 min), then adenylated (3′ end) by addition of A-Tailing Mix (37 °C for 30 min) and mixed to RNA index adapter and ligation mix (30 °C for 10 min). The final purification step was performed using AMPure XP beads (Beckman Coulter). Several rounds of PCR amplification were performed to enrich the cDNA fragments, prior to the purification of the PCR products. The libraries’ quality was assessed by checking the distribution of the fragments size using the Agilent bioanalyzer DNA 1000 (Agilent Technologies) and the libraries were quantified by RT-qPCR (KAPA Library Quantification Kit, Roche). The resultant cDNA libraries were paired-end sequenced (150 bp paired-end reads generated) on an Illumina HiSeq™ 4000 using the LIGAN-PM platform (Lille, France). Using CLC Genomics Workbench 11.0.1 software (Qiagen bioinformatics), a main component scatter plot (PCA, “Principal Component Analysis”) was performed based on the original expression values of the 10,061 genes expressed in the 18 gonadal samples before the differential expression analysis. Using the same metadata and software, a volcano plot was created to check for outlying samples of poor quality that need excluding from further analysis.

### Sequence and differential expression analysis (DEA)

All the analyses were conducted using the CLC Genomics Workbench 11.0.1 software (Qiagen bioinformatics). First, the Illumina reads were trimmed at the ends by quality scores, on the basis of the presence of ambiguous nucleotides (poly-N) (maximum value set at 2) using a modified version of the Mott algorithm (the quality limit was set at 0.05), as default sets on CLC Genomics Workbench software. The “RNA-Seq analysis” option in the software was used to map reads to the genome reference of *Crassostrea gigas* (genome v9 [63];; Assembly GCA_000297895.1; https://www.ncbi.nlm.nih.gov/assembly/GCF_000297895.1/) at the following settings: sequencing reads should be uniquely matched to the genome reference allowing up to two mismatches, without any insertion or deletion. The length fraction was at 0.8, minimum similarity was at 0.9 and the maximum number of hits pear read was 10. A differential gene expression analysis between different sexes and gametogenetic stages was performed using a model based on the negative binomial distribution. Only genes with a false discovery rate (FDR) < 0.05 and a fold change > 1.5 (*p*-value < 0.05) were considered as differentially expressed. All genes were functionally annotated by searching sequence similarities in the Swiss-Prot and Nr (NCBI, National Center for Biotechnology Information) databases using the CLC Genomics Workbench 11.0.1 software (Qiagen bioinformatics) with an e-value cut-off at 1e^− 5^. A Venn diagram was created to identify genes significantly differentially expressed between gametogenetic stages and sexes within *C. gigas* gonad transcriptome and was supplemented by a hierarchical cluster analysis performed using the “pheatmap” package from R. Finally, the results were imported into Blast2GO [17] and Gene Ontology (GO) terms at level 2 and an eggNOG annotation [35] with hit max at 20 were assigned.

### Quantitative real-time PCR (RT-qPCR)

In order to support the RNA-Seq data and to provide annual expression patterns for some relevant genes, RT-qPCR was undertaken. Seven genes that were differentially expressed based on DEA results, were selected for gene expression analysis in different stages of the gametogenetic cycle in both sexes. FoxL2 was also used here as a gold standard as its expression has already been studied in *C. gigas* by RT-qPCR ([45, 53]; GenBank Accession Number: FJ768956.1). Gene-specific primers were designed using Primer3 (v. 0.4.0) and custom synthesized by Eurogentec (France). Their sequences are listed in Table 4. The total RNA of twenty-four gonads (3 males and 3 females per stage; stages 0, 1, 2 and 3) were used for this experiment. They were first individually treated with DNAse I RQ1 (Promega) and purified with the NucleoSpin® RNA clean-up kit (Macherey-Nagel) in line with the manufacturer’s recommendations. Then cDNA was synthesized using M-MLV Reverse Transcriptase (Promega) with oligo (dT)_15_ Primer (Promega) in line with the manufacturer’s recommendations. The amplifications were conducted on a CFX96 Real-Time PCR Detection System (Biorad) using GoTaq® qPCR Master Mix (Promega). The cycling parameters were: 95 °C for 5 min, then 45 cycles of 95 °C for 15 s and 60 °C for 45 s. The PCR efficiency of each primer pair was determined based on a five-point standard curve generated from a two-fold dilution series (Additional File 3). Negative controls (total RNA not reverse-transcribed), blank controls (sterile water) and inter-plate controls were performed on each microplate. All PCR reactions were performed in technical triplicates. A melting curve analysis was performed to verify the specificity of each primer. The elongation factor 1-alpha gene (EF1α, GenBank Accession Number: AB122066) was used as a reference gene for gene expression normalization of targeted genes. The high stability of this housekeeping gene was previously identified by Dheilly et al. [21, 22] and was further confirmed in.

**Table 4:** List and sequences of primers used for RT-qPCR

Name or CGI of the corresponding gene	Name of the primer	Sequence of S and AS primers (5′- > 3′)
Elongation factor 1 α	EF1α S EF1α AS	ACCACCCTGGTGAGATCAAG ACGACGATCGCATTTCTCTT
FoxL2	FoxL2 S FoxL2 AS	AATATCAGGGATGGGCACAA TCCTTGGGTGCAGGAACTA
CGI_10,006,800	CGI_10,006,800 S1 CGI_10,006,800 AS1	GGTCTATCTTCGCTGGTTGC AGCAAATGCATGTTGATGGA
CGI_10,016,132	CGI_10,016,132 S2 CGI_10,016,132 AS2	GGGTCTAACGGGAAACCATT AACCAAAGTCACACCGGAAG
CGI_10,026,009	CGI_10,026,009 S2 CGI_10,026,009 AS2	ACCTCCTATGCCCATGACAG ACCATTGTCGGGCATTATGT
CGI_10,018,971	CGI_10,018,971 S1 CGI_10,018,971 AS1	TGCCACTGAACGAGTCTTTG GGTCCTCTTGCGTTCTTCTG
CGI_10,008,094	CGI_10,008,094 S2 CGI_10,008,094 AS2	CATGGCATTCAAAGGGAGAT TTCTCTTTAGTCCGCCTGGA
CGI_10,025,872	CGI_10,025,872 S2 CGI_10,025,872 AS2	CGACGGAGAATCCAGGACTA CAAGCGGTTGTAAGGTCCAT
CGI_10,028,666	CGI_10,028,666 S2 CGI_10,028,666 AS2	GACGACGGAGATCGCTAAAG TCTGGAACTGCGTGAGATTG

the present study by using the software BestKeeper^©^. Generally, reference genes with raw standard deviation (BKSTD) < 1 and with a coefficient of correlation (r) close to 1.0 are considered the most stable genes [39]. Relative gene expression was calculated using the 2-Δ∆CT method [43]. Statistical bilateral Wilcoxon tests were performed at each gametogenetic stage, in order to test whether the differences in gene expression were significant depending on the sex. Analyses were performed on R® (× 64 version, 3.2.2) with significance at *p* < 0.05.

## Supplementary Information

**Additional file 1 **Eighty-four genes identified in the *C. gigas* transcriptome by RNA-Seq Differential gene Expression Analysis (DEA): Name, NCBI gene ID, fold change (male vs female), *p*-value, FDR *p*-value, BLAST e-value, description. The 57 genes specifically differentially expressed between sexes in stage 0 or 3 are highlighted in bold.

**Additional file 2 **Statistical bilateral Wilcoxon tests performed for RT-qPCR experiments at each gametogenetic stage between sexes. Significance at *p* < 0.05.

**Additional file 3.** Primers used for RT-qPCR: list, sequences, number of bases, blasted name of the gene amplified, R^2^ and % of PCR efficiency.

## Data Availability

Raw reads obtained by RNA-Seq are available on NCBI (Sequence Read Archive *-* SRA) (BioProject ID: PRJNA660750 - https://www.ncbi.nlm.nih.gov/bioproject/PRJNA660750). The authors confirm that all data underlying the findings are fully available without restriction.

## Abbreviations

BKSTD: Raw STandard Deviation
Cg: *Crassostrea gigas*
Cg-FoxN5: *Crassostrea gigas* Forkhead Box N5
Cg-Sh3kbp1: *C. gigas* SH3 Domain Containing Kinase Binding Protein 1
CGI number: *Crassostrea gigas* Identification number
2-Δ∆CT: Delta-delta Cycle threshold
DEA: Differential gene Expression Analysis
DEG: Differentially Expressed Genes
Dsx/Dmrt1: Doublesex/Doublesex- and mab-3-related transcription factor 1
EF1α: Elongation Factor 1-alpha
eggNOG: evolutionary genealogy of genes Non-supervised Orthologous Groups
F: Female
FDR: False Discovery Rate
Fem: Feminizer
Fox L2: Forkhead box L2
FPKM: Fragments Per Kilobase of transcript per Million mapped reads
FSCN3: Fascin Actin-Bundling 3
Gata-4: Globin transcription factor 4
H-Y antigen: Male Histocompatibility antigen
lncRNA: long non-coding RNA
M: Male
MALRD1: MAM and LDL-receptor class A domain-containing protein 1-like
PML: ProMyelocytic Leukemia
Q20 and Q30: Phred Quality scores
SoxE/H: Sex-determining region Y box*-*containing E/H
St0, St1, St2, St3: Stage 0, stage 1, stage 2, stage 3
SUMO: Small Ubiquitin-like Modifier
TESK2: TEStis associated actin remodelling Kinase 2
Tra2: Transformer2
Waif1a: Wnt-activated inhibitory factor 1a
