# Supplementary material for: Gonadal transcriptomes associated with sex phenotypes provide potential male and female candidate genes of sex determination or early differentiation in Crassostrea gigas, a sequential hermaphrodite mollusc
Source: BMC Genomics. 2021 Aug 9;22:609. doi: 10.1186/s12864-021-07838-1 (PMC8353863; doi:10.1186/s12864-021-07838-1)
Supplement: Supplementary file 2 — Additional file 2 Statistical bilateral Wilcoxon tests performed for RT-qPCR experiments at each gametogenetic stage between sexes. Significance at p < 0.05. [file 12864_2021_7838_MOESM2_ESM.pdf]

p-value Wilcoxon	Stage 0	Stage 1	Stage 2	Stage 3
CGI 10011004	0.007898	0.000175	8.227e-05	8.227e-05
CGI 10006800	0.000931	0.000175	0.001024	0.002165
CGI 10016132	0.000631	0.000666	0.0004	0.02381
CGI 10026009	4.114e-05	0.000626	0.00041	0.001773
CGI 10018971	0.04009	0.000583	0.07487	0.01061
CGI 10008094	0.03636	0.007898	0.0004	8.227e-05
CGI 10025872	8.227e-05	4.114e-05	4.114e-05	0.00041
CGI 10028666	0.000666	0.05556	0.07552	1.00
